# Role of microRNA-21 in radiosensitivity in non-small cell lung cancer cells by targeting PDCD4 gene

**DOI:** 10.18632/oncotarget.15644

**Published:** 2017-02-23

**Authors:** Li-Peng Jiang, Chun-Yan He, Zhi-Tu Zhu

**Affiliations:** ^1^ Department of Radiation Oncology, First Affiliated Hospital of Liaoning Medical University, Jinzhou 121000, P.R. China; ^2^ Department of Prosthodontics, Second Affiliated Hospital of Liaoning Medical University, Jinzhou 121000, P.R. China; ^3^ Department of Oncology, First Affiliated Hospital of Liaoning Medical University, Jinzhou 121000, P.R. China

**Keywords:** microRNA-21, PDCD4, non-small cell lung cancer, radiotherapy, PI3K

## Abstract

This study aims to explore the effects of microRNA-21 (miR-21) on radiosensitivity in non-small cell lung cancer (NSCLC) by targeting programmed cell deanth 4 (PDCD4) and regulating PI3K/AKT/mTOR signaling pathway. Cancer tissues and adjacent normal tissues were collected from 97 NSCLC patients who received a standard radiotherapy regimen. TUNEL assay was applied to determine cell apoptosis in tissues. The qRT-PCR assay was used to detect the expressions of miR-21 expression and PDCD4 mRNA. The protein expressions of PDCD4 and PI3K/AKT/mTOR signaling pathway-related proteins were determined by Western blotting. Colony formation assay was used to observe the sensitivity to radiotherapy of NSCLC cells. Flow cytometry was adopted to testify cell apoptosis. Compared with adjacent normal tissues, miR-21 expression was significantly increased and the mRNA and protein expressions of PDCD4 were decreased in NSCLC tissues. Higher miR-21 expression was associated with attenuated radiation efficacy and shorter median survival time. PDCD4 was the target gene of miR-21. The miR-21 mimics and siRNA-PDCD4 decreased the sensitivity to radiotherapy and cell apoptosis of A549 and H1299 cells and activated PI3K/AKT/mTOR pathway. The sensitivity of A549 and H1299 cells was strengthened in the miR-21 inhibitors group and the PI3K/AKT/mTOR inhibitors group. The siRNA-PDCD4 could reverse the effects of miR-21 inhibitors on sensitivity to radiotherapy and cell apoptosis of NSCLC cells. Our findings provide strong evidence that miR-21 could inhibit PDCD4 expression and activate PI3K/AKT/mTOR signaling pathway, thereby affecting the radiation sensitivity of NSCLC cells.

## INTRODUCTION

Non-small cell lung cancer (NSCLC) accounts for about 85% of all lung cancer cases, which also include adenocarcinoma, large cell carcinoma, and squamous cell carcinoma [1]. Five-year survival rates can reach 20-30% in NSCLC patients after surgery [2]. Generally, elderly people are more likely to develop lung cancer than younger people, and males are at higher risk than that of females [3]. The main risk factor for NSCLC is smoking, to which 80% of lung cancer cases in males can be attributed [4]; other factors, such as occupational and environmental exposures, ionizing radiation, and genetic causes are also well-known risk factors for NSCLC [5]. At present, therapies for NSCLC include surgery, chemotherapy, and radiotherapy, and can be applied independently or jointly according to the range and progression of the disease [6]. Local and distant metastasis is a common clinical problem during the treatment of lung cancer, and is the primary contributor to poor prognosis in many patients [7]. Gene therapies for lung cancer, some of which have entered clinical trials, have developed rapidly in recent years [8]. Increasing the use of therapies that target and regulate gene expression and related molecular markers may improve NSCLC therapy [9].

MicroRNAs (miRs) are a recently discovered class of non-coding small RNA molecules approximately 22 nucleotides in length that play important roles in cell differentiation, proliferation, apoptosis, and metabolism by inhibiting target genes [10]. The miR-21 is up-regulated in multiple solid tumors, including lung cancer, breast cancer, and pancreatic cancer, and is overexpressed in most cancer tissues, suggesting a close relationship between miR-21 expression and tumor development [11]. Differences in miR expression are related to the efficacy of radiotherapy in NSCLC, and miRs may therefore serve as biomarkers for predicting the efficacy of radiotherapy [12]. For instance, miR-25 was reported to directly regulate B-cell translocation gene 2 expressions, leading to modulation on sensitivity of radiotherapy in NSCLC cells [13]. Human programmed cell death 4 (PDCD4) gene was mapped in chromosome 10q24, but its function has not been well defined [14]. MiR-21 regulates the expression of PDCD4 mRNA and protein, and thus reduces the sensitivity of lung cancer to radiotherapy [15, 16]. The PDCD4 gene, which is frequently expressed in normal tissues and functions by affecting gene translation and transcription, was initially discovered for its role in cell apoptosis [17]. In addition, a previous study reported that miR-21 is highly expressed in patients with NSCLC and inhibition of miR-21 expression reduces proliferation, migration, and invasion of A549 cells by up-regulating PDCD4 expression [18]. Therefore, we hypothesized that the regulation of miR-21 in PDCD4 gene may relate to the sensitivity of radiotherapy in NSCLC. Furthermore, phosphatidylinositol-3-kinase/protein kinase B/mammalian target of Rapamycin (PI3K/AKT/mTOR) pathway was reported to implicate in cell survival, proliferation and angiogenesis [19]. And the therapeutic potential of combination strategy for mTOR inhibitors with conventional chemotherapy in anti-microtubule agents, other molecular targeting agents, as well as radiotherapy was highlighted in hepatocellular carcinoma [20]. In this regards, the possible role of PI3K/AKT/mTOR signaling pathway is also investigated to explore the possible mechanism of sensitivity of radiotherapy in NSCLC. Therefore, we explore the effects of miR-21 on sensitivity to radiotherapy in NSCLC by targeting PDCD4 and regulating PI3K/AKT/mTOR signaling pathway.

## RESULTS

### Comparisons of cell apoptosis and miR-21 expression and *PDCD4* mRNA expression in NSCLC tissues and adjacent normal tissues before and after radiotherapy

**Figure 1:**
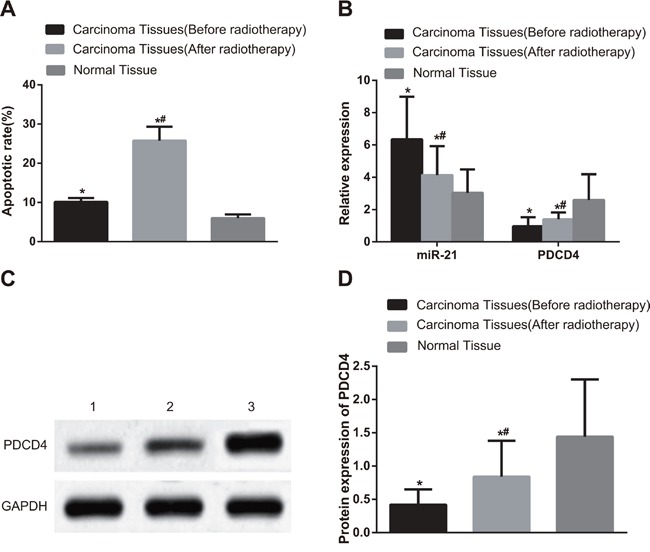
Comparisons of cell apoptosis and the miR-21 expression, PDCD4 mRNA and protein expressions in NSCLC and adjacent normal tissues before and after radiotherapy Note: **A**. Comparisons of apoptotic index between NSCLC tissues and adjacent normal tissues before and after radiotherapy; **B**. Comparisons of the miR-21 expression and PDCD4 mRNA expression between NSCLC tissues and adjacent normal tissues before and after radiotherapy; **C**. The protein expression of PDCD4 detected by Western blotting; 1, NSCLC tissues (before radiotherapy); 2, NSCLC tissues (after radiotherapy); 3, adjacent normal tissues (before radiotherapy); **D**. Comparisons of the PDCD4 protein expression between NSCLC tissues and adjacent normal tissues before and after radiotherapy; *, compared with adjacent normal tissues, *P* < 0.05; #, compared with those before radiotherapy, *P* < 0.05; NSCLC, non-small cell lung cancer; PDCD4, programmed cell death 4; GAPDH, glyceraldehyde-3-phosphate dehydrogenase; miR-21, microRNA-21.

As shown in Figure 1A, compared with adjacent normal tissues, the apoptotic index (AI) values of NSCLC tissues were significantly elevated before and after radiotherapy (*P* < 0.001). In NSCLC tissues, the AI value after radiotherapy was higher than that before radiotherapy (*P* < 0.001). The miR-21 expression in NSCLC tissues before and after radiotherapy (before, 6.35 ± 2.64; after, 4.14 ± 1.79) was higher than that in adjacent normal tissues (3.04 ± 1.45) (Figure 1B, both *P* < 0.05). In contrast, *PDCD4* mRNA expression in NSCLC tissues before and after radiotherapy (before, 0.96 ± 0.57; after, 1.47 ± 0.32) was lower than that in adjacent normal tissues (2.60 ± 1.59) (both *P* < 0.05). The miR-21 expression in NSCLC tissues after radiotherapy was remarkably decreased compared with that before radiotherapy, while *PDCD4* mRNA expression in NSCLC tissues after radiotherapy was elevated in comparison with that before radiotherapy (both *P* < 0.05). PDCD4 protein expression in NSCLC tissues before and after radiotherapy (before, 0.42 ± 0.23; after, 0.84 ± 0.54) was lower than that in adjacent normal tissues (1.44 ± 0.86) (Figure 1C & 1D, both *P* < 0.05). PDCD4 protein expression in NSCLC tissues after radiotherapy was elevated in comparison with that before radiotherapy (both *P* < 0.05).

### Correlations of miR-21 expression and *PDCD4* mRNA and protein expressions with radiotherapy efficacy of NSCLC patients

After radiotherapy, there were 14 cases of complete remission (CR), 44 cases of partial remission (PR), 23 cases of stable disease (SD), and 16 cases of progressive disease (PD). The effective rate (CR + PR) was 59.8%. As shown in Table 1, no significant difference was revealed concerning miR-21 expression and mRNA and protein expressions of PDCD4 between the CR group and the PR group and between the SD group and the PD group (both *P* > 0.05). The CR and PR groups exhibited lower miR-21 expression and higher mRNA and protein expressions of PDCD4 than those in the SD and PD groups (all *P* < 0.05).

**Table 1:** Correlations the miR-21 expression, PDCD4 mRNA and protein expression with sensitivity to radiotherapy of NSCLC patients

Group	N	miR-21	PDCD4 mRNA	PDCD4 Protein
CR	14	3.32 ± 1.29	1.65 ± 0.53	1.19 ± 0.72
PR	44	3.63 ± 1.36	1.59 ± 0.24	1.07 ± 0.34
SD	23	4.74 ± 2.17^*#^	1.27 ± 0.19	0.47 ± 0.50*^#^
PD	16	5.42 ± 1.76^*#^	1.25 ± 0.15	0.44 ± 0.28^*#^

Note: miR-21, microRNA-21; PDCD4, programmed cell death 4; NSCLC, non-small cell lung cancer; CR, complete remission; PR, partial remission; SD, stable disease; PD, progressive disease;* indicates when comparing with the ineffective group, *P* < 0.05; ^#^ indicates when comparing with the ineffective group, *P* < 0.05.

### Effects of miR-21 on long-term efficacy of patients after radiotherapy

**Figure 2:**
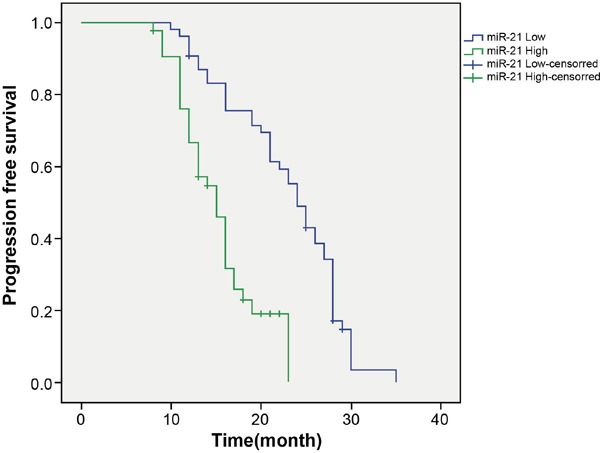
Comparison of progression free survival between the low miR-21 expression group and the high miR-21 expression group Note: miR-21, microRNA-21.

Patients were classified into the low miR-21 expression group (miR-21 ≤ 4.23) and the high miR-21 expression group (miR-21 > 4.23). In the high miR-21 expression group, 4 patients died among the 43 cases (4/43, 9.30%) with a median progression free survival (PFS) of 15 months. In the low miR-21 expression group, 2 contacts were lost among the 54 cases (2/54, 3.70%) with a PFS of 24 months. The PFS Kaplan-Meier curve of the two groups was drawn in Figure 2. By log-rank test, the PFS of the high miR-21 expression group was reduced compared to the low miR-21 expression group (*P* < 0.05). The univariate analysis revealed that no statistical difference was found in terms of age, gender, and smoking history on the effects of long-term efficacy (Table 2, *P* > 0.05), whereas, tumor-node-metastasis (TNM) stage and lymph node metastasis were significantly associated with long-term efficacy (*P* < 0.05).

**Table 2:** Univariate analysis of median progression free survival of the high miR-21 expression group and the low miR-21 expression group

Variable	Low miR-21 expression group	High miR-21 expression group	*P*
Age (years)			
≥ 60	32	28	0.555
< 60	22	15	
Gender			
Male	30	26	0.627
Female	24	17	
Smoking history			
Yes	41	37	0.304
No	13	6	
Lymph node metastasis			
Yes	35	37	0.018
No	19	6	
TNM stage			
I stage	11	2	0.030
II stage	15	12	
III stage	15	9	
IV stage	13	20	

Note: TNM, tumor-node-metastasis; miR-21, microRNA-21.

### Comparisons of the miR-21 expression, PDCD4 mRNA and protein expressions in each group after cell transfection

**Figure 3:**
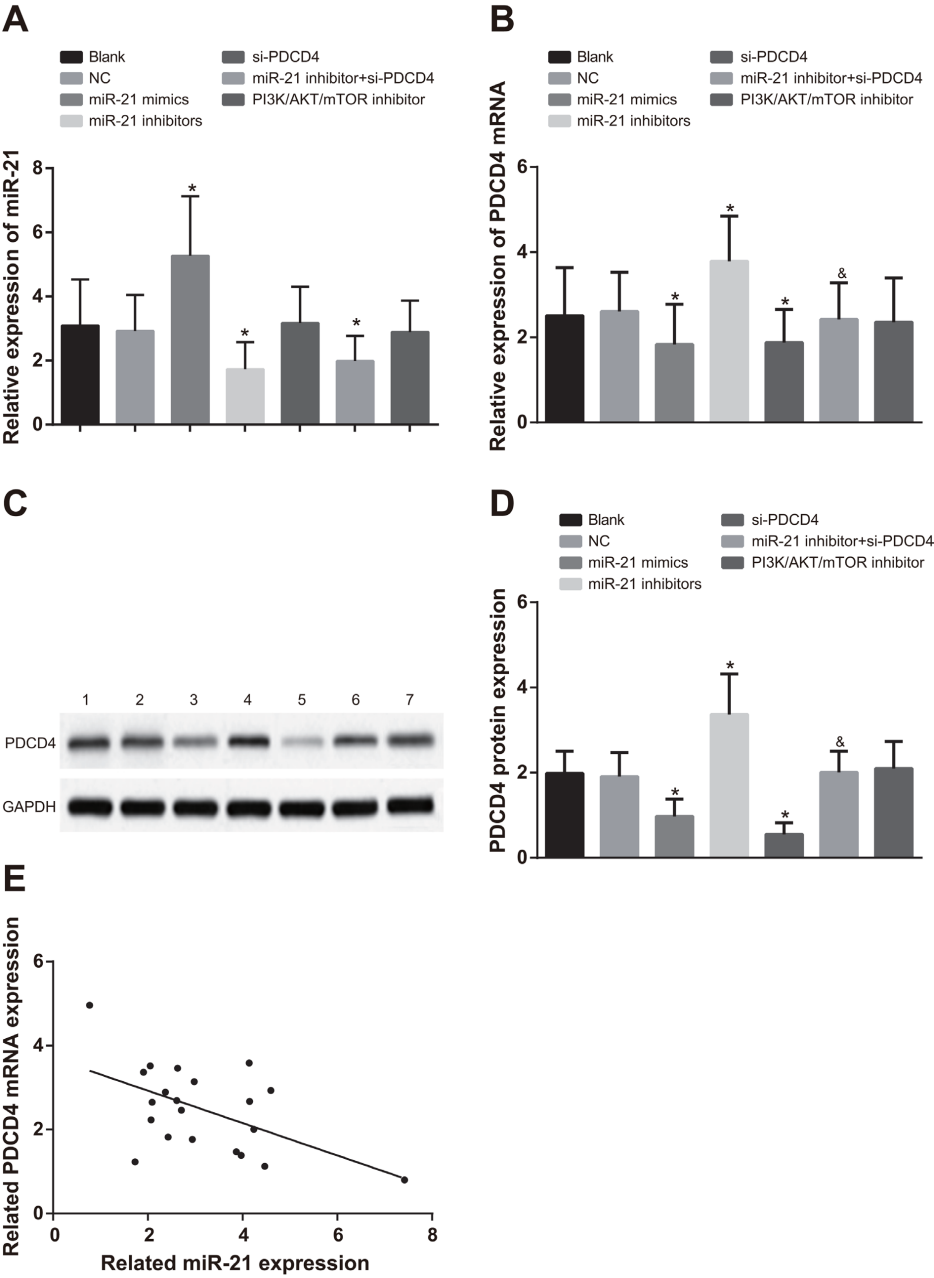
Comparisons of miR-21 expression and mRNA and protein expressions of PDCD4 among different groups in A549 cells Note: *, compared with the blank group, *P* < 0.05; ^&^, compared with the miR-21 inhibitors group, *P* < 0.05; 1, blank group; 2, negative control group; 3, miR-21 mimics group; 4, miR-21 inhibitors group; 5, si-PDCD4 group; 6, miR-21 inhibitors + si-PDCD4 group; 7, PI3K/AKT/mTOR inhibitors group; PDCD4, programmed cell death 4; miR-21, microRNA-21.

**Figure 4:**
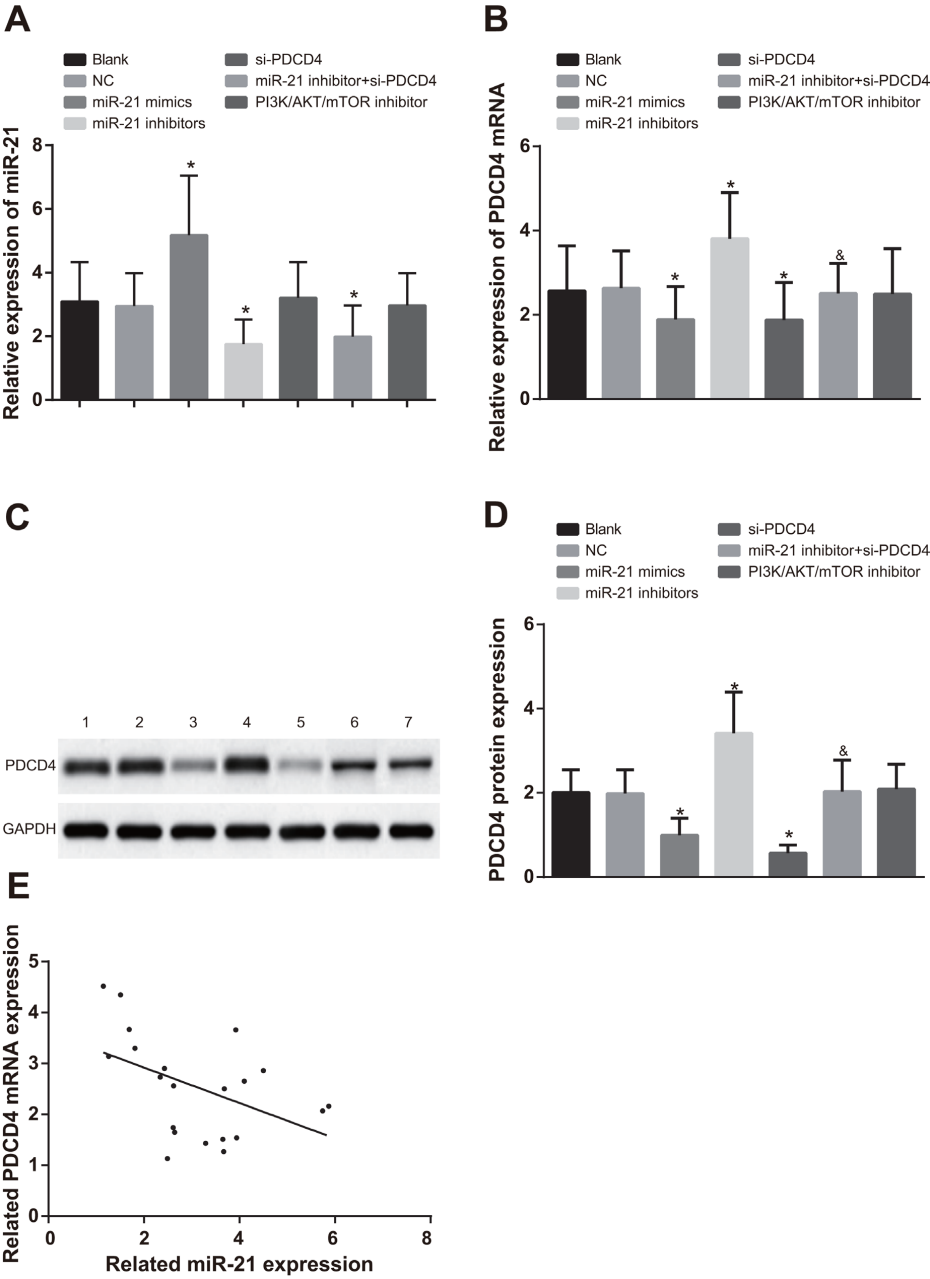
Comparisons of miR-21 expression and mRNA and protein expressions of PDCD4 among different groups in H1299 cells Note: *, compared with the blank group, *P* < 0.05; ^&^, compared with the miR-21 inhibitors group, *P* < 0.05; 1, blank group; 2, negative control group; 3, miR-21 mimics group; 4, miR-21 inhibitors group; 5, si-PDCD4 group; 6, miR-21 inhibitors + si-PDCD4 group; 7, PI3K/AKT/mTOR inhibitors group; PI3K/AKT/mTOR, phosphatidylinositol-3-kinase/protein kinase B/mammalian target of Rapamycin; PDCD4, programmed cell death 4; miR-21, microRNA-21.

In A549 cells, compared with the blank group and the NC group, miR-21 expression in the miR-21 mimics group was evidently increased, while significantly decreased in the miR-21 inhibitors and miR-21 inhibitors + si-PDCD4 groups (Figure 3A, all *P* < 0.05). There was no significant difference in miR-21 expression in the si-PDCD4 group and the PI3K/AKT/mTOR inhibitors group in contrast to the blank and NC groups (all *P* > 0.05). In comparisons to the blank and NC groups, PDCD4 mRNA and protein expressions were decreased in both the miR-21 mimics and si-PDCD4 groups, but increased in the miR-21 inhibitors group (Figure 3B & 3D, all *P* < 0.05). There was no significant difference in PDCD4 mRNA and protein expressions in the PI3K/AKT/mTOR inhibitors group compared to the blank and NC groups (all *P* > 0.05). PDCD4 mRNA and protein expressions were inhibited in the miR-21 inhibitors + si-PDCD group in comparison to the miR-21 inhibitors group (*P* < 0.05). The observations in H1299 cells were similar with that in A549 cells (Figure 4). The miR-21 expression was negatively associated with PDCD4 mRNA expressions and overexpression of miR-21 was able to suppress PDCD4 expression (Figure 3E & 4E).

### Targeting relationship between miR-21 and PDCD4

**Figure 5:**
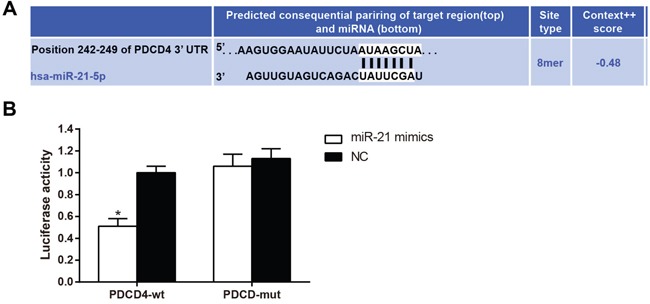
PDCD4 is the target gene of miR-21 Note: **A**., the binding site of miR-21 with PDCD4 in 3′-UTR as predicted by TargetScan; **B**., luciferase activity between the miR-21 mimics and NC groups; In cells of the PDCD4 group, transfection with miR-21 decreased the luciferase activity as compared with the NC group; *, compared with the control group, *P* < 0.05; PDCD4, programmed cell death 4; miR-21, microRNA-21; NC, negative control.

Using the TargetScan database as a reference, PDCD4 was identified as a potential target gene of miR-21 (Figure 5A). Dual luciferase reporter gene assays were used to confirm this result. The luciferase signal in cells transfected with both miR-21 and PDCD4-3′-UTR decreased by about 49% as compared to the other groups (Figure 5B, all *P* < 0.05). Luciferase signals did not decrease in any of the groups transfected with PDCD4 mut-3′-UTR mutants (all *P* > 0.05). These results indicated that miR-21 binds to the 3′ UTR region of *PDCD4*, inhibiting its transcription and thus regulating its expression. PDCD4 therefore was likely a direct target gene of miR-21.

### Comparisons of the expressions of PI3K/AKT/mTOR signaling pathway-related proteins in each group after radiotherapy

**Figure 6:**
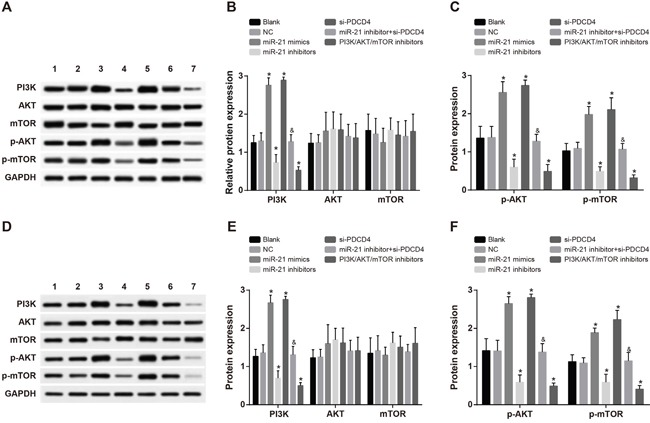
Comparisons of the expressions of PI3K/AKT/mTOR signaling pathway-related proteins among different groups in A549 and H1299 cells after radiotherapy Note: 1, blank group; 2, negative control group; 3, miR-21 mimics group; 4, miR-21 inhibitors group; 5, si-PDCD4 group; 6, miR-21 inhibitors + si-PDCD4 group; 7. PI3K/AKT/mTOR inhibitors group; *, compared with the blank group, *P* < 0.05; ^&^, compared with the miR-21 inhibitors group, *P* < 0.05; PI3K/AKT/mTOR, phosphatidylinositol-3-kinase/protein kinase B/mammalian target of Rapamycin; PDCD4, programmed cell death 4; miR-21, microRNA-21.

As shown in Figure 6, there were no significantly differences in expressions of AKT and mTOR among all the transfected groups in both A549 and H1299 cells (all *P* > 0.05). No significant difference was found regarding expressions of PI3K, p-AKT and p-mTOR between the blank group and the NC group (all *P* > 0.05). Compared with the blank group, the expressions of PI3K, p-AKT and p-mTOR were remarkably increased in both the miR-21 mimics group and the si-PDCD4 group, but decreased in the miR-21 inhibitors group and the PI3K/AKT/mTOR inhibitors group (all *P* < 0.05). The miR-21 inhibitors group had relative lower expressions of PI3K, p-AKT and p-mTOR in comparison to the miR-21 inhibitors + si-PDCD4 group (all *P* < 0.05). Those results suggested that miR-21 inhibitors could suppress the activation of PI3K/AKT/mTOR signaling pathway and si-PDCD4 could reverse this suppression.

### Effect of miR-21 on radiosensitivity of NSCLC cells in each group

**Figure 7:**
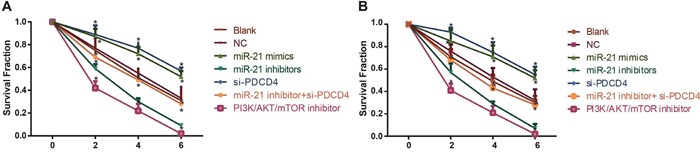
Comparisons of survival fraction among different groups under different radiation doses Note: **A**. The clone formation curve for different groups in A549 cells; **B**. the clone formation curve for different groups in H1299 cells; *, compared with the blank group, *P* < 0.05; ^&^, compared with the miR-21 inhibitors group, *P* < 0.05; PDCD4, programmed cell death 4; miR-21, microRNA-21.

The results for colony formation assay were presented in Figure 7. There were no significant differences in survival fraction (SF) of H1299 and A549 cells in the blank and NC groups at 0 Gy, 2 Gy and 4 Gy (all *P* > 0.05). The SF in the miR-21 mimics group and the si-PDCD4 group were decreased compared with the blank group and the NC group (all *P* < 0.05) in a dosage-dependent manner. The SF in the miR-21 inhibitors group and the PI3K/AKT/mTOR inhibitors group were lower in comparison with the blank and NC groups in a dosage-dependent manner (all *P* < 0.05). Compared with the miR-21 inhibitors group, the SF in the miR-21 inhibitors + si-PDCD4 group was elevated (*P* < 0.05). Those results suggested that miR-21 inhibitors could enhance the radiosensitivity in A549 and H1299 cells, but the application of si-PDCD4 could reverse the decreased radiosensitivity induced by miR-21 inhibitors.

### Comparisons of cell apoptosis of NSCLC cells in each group after radiotherapy

**Figure 8:**
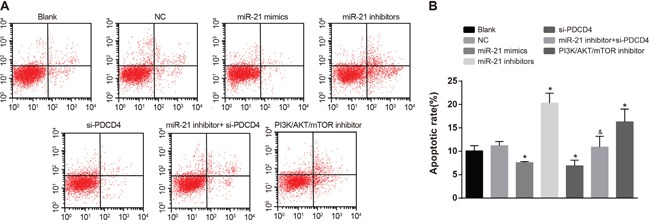
Cell apoptosis among different groups in A549 cells detected by flow cytometry Note: *, compared with the blank group, *P* < 0.05; ^&^, compared with the miR-21 inhibitors group, *P* < 0.05; PDCD4, programmed cell death 4; PDCD4, programmed cell death 4; miR-21, microRNA-21.

**Figure 9:**
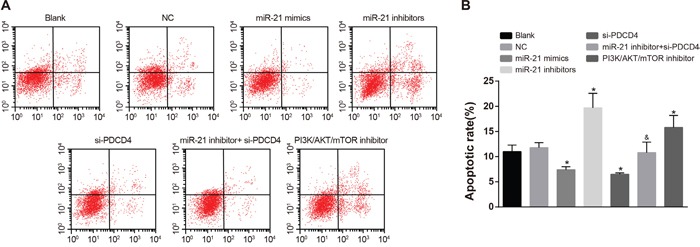
Cell apoptosis among different groups in H1299 cells detected by flow cytometry Note: *, compared with the blank group, *P* < 0.05; ^&^, compared with the miR-21 inhibitors group, *P* < 0.05; PDCD4, programmed cell death 4; miR-21, microRNA-21.

As showed in Figure 8, there was no significant difference in apoptosis rate between the NC group and the blank group in A549 cells. In comparisons with the blank group, the apoptosis rate in both the miR-21 mimics group and the si-PDCD4 group was decreased, while the apoptosis rate in the miR-21 inhibitors group and the PI3K/AKT/mTOR inhibitors group was increased (all *P* < 0.05). The miR-21 inhibitors + si-PDCD4 group had relative lower apoptosis rate compared with the miR-21 inhibitors group (*P* < 0.05). The observations in A549 cells were the same with those in H1299 cells (Figure 9). Above results showed that miR-21 inhibitors could elevate the apoptosis rate in both A549 and H1299 cells, while si-PDCD4 could decrease the promotion of apoptosis induced by miR-21 inhibitors.

## DISCUSSION

High miR-21 expression and low PDCD4 mRNA expression in NSCLC tissues correlate strongly with disease occurrence, invasion, and metastasis, and therefore predict poor prognosis in NSCLC patients [21]. Additionally, upregulation of PDCD4 and downregulation of miR-21 inhibits NSCLC proliferation and migration [18]. Here, we explore the specific mechanisms by which miR-21 regulates the expression of PDCD4 and subsequently affects the efficacy of radiotherapy in NSCLC. Our results may facilitate clinical diagnosis and prognosis evaluation for NSCLC patients.

In this study, miR-21 expression was higher in NSCLC tissues than in normal adjacent tissues, and this elevated expression was associated with reduced efficacy of radiotherapy. MiRs are involved in the regulation of cell development, differentiation, proliferation, and apoptosis, and are closely related to the occurrence and development of many diseases [22]. MiR-21 is an oncogenic miR in human malignant tumors, and it is the only miR known to be highly expressed in almost all solid tumors [23, 24]. MiR-21 regulates cell differentiation, proliferation, and apoptosis by regulating target genes, and it is also involved in tumor growth, invasion, and metastasis [25, 26]. The inhibition of miR-21 suppresses proliferation and accelerates apoptosis in cancer cells and renders them more sensitive to chemotherapy [27]. Abnormal miR-21 expression affects the efficacy of various chemotherapy drugs, including gemcitabine, hycamtin, 5-fluorouracil, temozolomide, and taxotere [28, 29]. MiR-21 is overexpressed in many solid tumors, including colon cancer, breast cancer, and gastric cancer, and high miR-21 expression correlates with disease progression and poor prognosis in patients [30–32]. Tian *et al*. showed that altered miR-21 expression is related to NSCLC occurrence, development, and survival analysis indicated that miR-21 expression is negatively correlated with NSCLC progression [33]. MiR-21 is also an important miRNA regulating radiosensitivity of human cancer cells [34, 35]. MiR-21 enhances radioresistance in NSCLC through regulation of phosphatase and tensin homolog deleted on chromosome ten (PTEN) [36].

Here, we found that miR-21 targets PDCD4, and elevated miR-21 expression inhibited PDCD4 expression. Moreover, miR-21 inhibitors can enhance the sensitivity to radiotherapy and reduce cell apoptosis in both A549 and H1299 cells, while those effects can be reversed by the application of si-PDCD4. As a result, miR-21 decreased sensitivity to radiotherapy and enhanced the ability of cancer cells to resist apoptosis, thus reducing the efficacy of radiotherapy. PDCD4, a novel tumor suppressor gene, is involved in apoptosis, and deletions in or reduced expression of PDCD4 have been found in many human malignant tumor tissues and cells [37]. By binding to the translation initiation factor eIF4A (eukaryotic initiation factor-4A), PDCD4 inhibits the translation of proteins and accelerates apoptosis [38]. It also inhibits tumor growth, invasion, and metastasis by activating and regulating the transcription activator protein AP-1 and matrix metalloproteinase 2 (MMP-2) [39, 40]. Zhu *et al*. also confirmed that miR-21 could modulate PDCD4 expression at the translational and post-transcriptional levels in HepG2 cells, enhance phosphorylation of c-Jun protein, and activate AP-1-dependent transcription of MMP-2 as well as MMP-9, thus promoting migration and invasion of hepatocellular carcinoma cell lines [41]. PDCD4 expression in poorly-differentiated colorectal cancer, gastric cancer, and pancreatic cancer was lower than in highly-differentiated cancers, and deletions in or decreased expression of PDCD4 correlated with poor prognosis [37]. PDCD4 expression was negatively associated with miR-21 levels in gastric cancer [14]. By inhibiting PDCD4, miR-21 acts as an anti-apoptotic factor and promotes tumor cell transformation [42]. Asmlgani *et al*. found that miR-21 inhibits apoptosis and promotes proliferation in colorectal cancer tumor cells by regulating PDCD4 [43]. The miR-21 also promotes tumor cell invasion and metastasis in metastatic breast cancer MDA-MB-231 cells by inhibiting the expression of PDCD4 [44]. The study of Chao *et al*. found that miR-21 knockdown in glioblastoma cells could increase the expression of PDCD4 and hMSH2, which further enhance the radiation sensitivity of cancer cells [35]. Similarly, in our study, miR-21 promoted NSCLC progression by inhibiting PDCD4. To further explore the mechanism of miR-21 decreasing the sensitivity of radiotherapy in NSCLC via targeting PDCD4, the expressions of PI3K/AKT/mTOR signaling pathway related proteins were detected after radiotherapy. Our results suggested that miR-21 inhibitor can suppress the activation of PI3K/AKT/mTOR signaling pathway and si-PDCD4 can reverse this suppression. As proved that miR-21 inhibitor can increase apoptosis rate and enhance the sensitivity of radiotherapy in NSCLC A549 and H1299 cells, it is speculated that miR-21 inhibitor can accelerate cell apoptosis and enhance sensitivity of radiotherapy in NSCLC cells by suppressing PDCD4 and inactivating the PI3K/AKT/mTOR signaling pathway.

In conclusion, miR-21 could inhibit PDCD4 expression and affect sensitivity to radiotherapy in NSCLC through activating PI3K/AKT/mTOR signaling pathway. In addition, miR-21 inhibitor enhances cell apoptosis in NSCLC and si-PDCD4 could reverse those effects of miR-21. Therefore, miR-21 might be a potential target for enhancing the clinical radiosensitivity in NSCLC in the future. Such research may ultimately lead to novel strategies for the clinical treatment of NSCLC. It should be borne in mind that it is possible that other miRs may also target PDCD4, however, the limitation in time and budget restricted us from conducting any other detection. Therefor further analysis of PDCD4 with other miRs can be served as a future topic in our further experiments.

## MATERIALS AND METHODS

### Subjects

Cancer tissues and adjacent normal tissues (≥ 5 cm away from tumor tissue) were collected from 97 NSCLC patients at the First Affiliated Hospital of Liaoning Medical University between February 2013 and August 2015. Fifty-six patients were males and 41 were females, and patient ages ranged from 38 to 81 years with a mean age of 62.73 ± 9.44 years. Fifty-three cases were identified as squamous carcinoma and 44 cases as adenocarcinoma during pathological examinations. And 60 cases exhibited lymph node metastasis (LNM), while 37 cases did not. TNM stages were determined according to the Union for International Cancer Control standard [45]. There were 14 cases at stage I, 27 cases at stage II, 21 cases at stage III, and 35 cases at stage IV. All patients had normal cardiac and pulmonary function and none received chemotherapy. Tissue samples were quickly transferred to liquid nitrogen and subsequently stored at -80°C until use. This study was approved by the Ethics Committee of the First Affiliated Hospital of Liaoning Medical University. All patients and their families signed informed consent forms.

### Treatment regimen

Before treatment, all patients underwent a computed tomography (CT) scan. Different treatment strategies were developed taking into consideration tumor location, tumor size, and other relevant factors. Patients received three-dimensional conformal radiation therapy after surgery at 2 Gy per treatment 5 times per week for a total radiation dose of about 50~70 Gy over a period of 8 weeks. Radiotherapy efficacy was judged according to the WHO standard [46]. Patients were then categorized into the effective (complete remission [CR] + partial remission [PR]) and ineffective (stable disease [SD] + and progression disease [PD]) groups.

### Follow-up

Follow-up visits were conducted through telephone, consultation to outpatient department and reference to medical records, which was terminated in June 2016 after 3~36 months. In total, 6 cases were lost, resulting in a follow-up rate of 93.81%. The PFS of patients were recorded, which commenced from the treatment and ended in NSCLC recurrence, cancer-related death or termination of follow-up.

### Terminal deoxynucleotidyl transferase-mediated dUTP nick-end labelling (TUNEL) assay

After routine dewaxing and dehydration, tissue samples were blocked by hydrogen peroxide and incubated in microwave for 20 min. Terminal Deoxynucleotidyl Transferase (TDT) and biod UTP (Roche Diagnostics GmbH, Mannheim, Germany) were added for incubation at 37°C for 1 h. Biotin-labeled horseradish peroxidase was then supplemented for incubation at 37°C for 1 h. Samples were then developed with 3,3′-diaminobenzidine tetrahydrochloride (DAB), stained, dehydrated and mounted with neutral resin. Sections with positive expressions of apoptosis cells were considered as positive control. Phosphate buffer solution (PBS) was used as primary antibody for negative control. Apoptotic cells were recognized that were stained into brown in nucleus. And apoptotic cells in randomized 10 low-magnification fields were selected and calculated for AI.

### Cell transfection and grouping

The A549 and H1299 NSCLC cell lines were provided by the Shanghai Institute of Biochemistry and Cell Biology of the Chinese Academy of Sciences (Shanghai, China). A549 and H1299 cells were seeded on 6-well plates at a density of 5 × 10^4^ cells/mL. The cells were cultured in Roswell Park Memorial Institute (RPMI)-1640 medium containing 10% imported fetal bovine serum, 100 U/mL penicillin, and 100 mg/L streptomycin. The cells were cultured at 37°C in a saturated humidity atmosphere containing 5% carbon dioxide. During cell passage, cells were trypsinized with 0.125% trypsin [including 0.1% ethylenediaminetetraacetic acid (EDTA)]. A549 and H1299 cells in the logarithmic growth phase were collected and assigned into 5 groups: (1) blank group; (2) negative control (NC) group; (3) miR-21 mimics group; (4) miR-21 inhibitors group; (5) si-PDCD4 group; (6) miR-21 inhibitors + si-PDCD4 group; and (7) PI3K/AKT/mTOR inhibitors group (penicillin as inhibitor). The si*-*PDCD4, miR-21 mimics, miR-21 inhibitors, and RNA molecule in the NC group were synthesized by Life Technologies Corp. (Grand Island, New York, USA). The primer sequence of miR-21 inhibitor was 5′-GTCGTATCCAGTGCAGGGTCCGAGGTATTCGCACTGCATAC GACTCAACA -3′. The miR-21 inhibitor was transfected after passage of 24 h, when cells reached 60~70% confluence. Following transfection of 24 h, the total RNA was extracted for further experiment.

### Quantitative real-time polymerase chain reaction (qRT-PCR)

The RNeasy Mini Kit (Qiagen, Hilden, Germany) was used according to the manufacturer's protocol to extract total RNA from tumor tissues and adjacent normal tissues. A260/A280 ratio and RNA concentration were measured using a NanoDrop ultraviolet spectrophotometer. Reverse transcription of tissue RNA into cDNA was performed using a reverse transcription kit from Promega (Promega Corp., Madison, Wisconsin, USA). The quantitative fluorescence PCR kit was purchased from Shanghai GenePharma Co., Ltd. (Shanghai, China). The qRT-PCR reaction was carried out using ABI 7500 (Applied Biosystems, Oyster Bay, New York, USA). The reaction volume was 20 μL, and the reaction conditions were as follows: 3 min at 95°C followed by 12 s at 95°C and 50 s at 62°C for 40 cycles. U6 and β-actin were used as internal controls for the quantification of miR-21 and PDCD4, respectively. Primers used in the experiments were listed in Table 3. The 2^−ΔΔCT^ method was used to calculate relative expressions.

**Table 3:** Primer sequences for quantitative real-time polymerase chain reaction

Gene	Primer sequence
miR-21	F: 5′-ACGTTGTGTAGCTTATCAGACTG-3′
	R: 5′-AATGGTTGTTCTCCACACTCTC-3′
U6	F: 5′-ATTGGAACGATACAGAGAAGATT-3′
	R: 5′-GGAACGCTTCACGAATTTG-3′
PDCD4	F: 5′-ATGTGGAGGAGGTGGATGTG-3′
	R: 5′-TGGTGTTAAAGTCTTCTCAAATGC-3′
β-actin	F: 5′-GTGGGGCGCCCCAGGCACCA-3′
	R: 5′-CTCCTTAATGTCACGCACGATTT-3′

Note: F, forward; R, reverse; miR-21, microRNA-21; PDCD4, programed cell death 4.

### Western blotting of PDCD4 protein

Tissue sample cells and transfected cells were washed with PBS, added to 100 μL of cell lysis solution, and incubated at 4°C for 30 min. The lysate was centrifuged at 12000 × g for 10 min, and the protein concentration of the supernatant was measured using the Bradford method. Twelve percent sodium dodecyl sulfate polyacrylamide gel electrophoresis (SDS-PAGE) was performed with a total protein content of 50 μg in each lane, and proteins were electro-transferred onto a polyvinylidenefluoride membrane. After incubation with 5% skim milk in a closed container at room temperature for 1 h, 1: 200 dilution of mouse anti-human PDCD4 antibody and glyceraldehyde-3-phosphate dehydrogenase (GAPDH) monoclonal antibody were added to the mixture followed by incubation overnight at 4°C. A 1: 10,000 dilution of IRDyeTM800DX-labeled sheep anti-mouse IgG was added to the mixture after the membrane was washed. Antibodies were purchased from Upstake (USA). After 1 h of incubation in dark at room temperature, the membrane was fully washed and placed in an Odyssey (Li-cor Biosciences, Lincoln, NE, USA) dual-color infrared laser imaging system for direct image scanning. The integrated optical density of each band was calculated, and relative expression was determined using the ratio of the integrated optical density of the target protein band to that of the GAPDH internal control band.

### Dual luciferase reporter gene assay

Target gene analysis was performed using the TargetScan database in order to determine whether PDCD4 was a direct target of miR-21. The full-length PDCD4 gene 3′ UTR region was cloned and amplified. The PCR product was cloned into multiple cloning sites downstream of a luciferase gene in a pmirGLO (Promega Corp., Madison, Wisconsin, USA) vector. The predicted miR-21 binding sites in the target gene were subjected to site-directed mutagenesis, and the *Renilla* luciferase-expressing vector PRL-TK (Takara Holdings Inc., Kyoto, Japan) was used as an internal control to adjust for differences in cell numbers and transfection efficiency. Both miR-21 and negative control were transfected into A549 cells with the luciferase vector, and the dual luciferase assay was performed according to the protocols provided by Promega Corp. (Madison, Wisconsin, USA).

### Colony formation assay

Cells in the logarithmic growth phase were washed with PBS twice for cell suspension. After calculated by cell counting method, the cells were diluted into appropriate density and inoculated into 60-mm-diameter plates based on different irradiation doses. Three duplicated wells were set for each irradiation dose. A549 and H1299 cells in the blank group, NC group, miR-21 mimics group, miR-21 inhibitors group, si-PDCD4 group and miR-21 inhibitors + si-PDCD4 group received 0 Gy, 2 Gy, 4 Gy and 6 Gy irradiation, followed by incubation for 10~14 days. During the incubation, the culture medium can be replaced for 1-2 times. In the 14^th^ day of incubation, cells were washed in PBS, fixed in methanol for 20 min and stained with 0.1% crystal violet for 15 min. After the medium were dried, low-magnification microscope was used to calculate the clone numbers of the colonies that had more than 50 cells. Plating efficiency (PE) and survival fraction (SF) were also calculated based on the following formula: PE = (clone numbers in the blank group/number of inoculated cells in the blank group) × 100%, SF = clone numbers / (number of inoculated cells × PE).

### Flow cytometry

A549 and H1299 cells in different transfection groups were seeded into 96-well plates in a concentration of 2 × 10^4^/mL. Each well received 100 μL of cells and was cultured for 24 h. The cells received 4 Gy of X ray radiotherapy after attaching to the well surface. After radiotherapy for 48 h, annexin V–fluoroscein isothiocyanate (FITC)/propidium iodide (PI) double staining kit (Keygen biotechnology, Jiangsu, China) was used to detect cell apoptosis rate. Cells were obtained and washed twice with PBS, and then centrifuged for 5 min at 1900 × g. The supernatant was discarded, and annexin V-FITC/PI staining solution was added into 100 μL of cell suspension, which was mixed and incubated in dark for 15 min at room temperature. The flow cytometry measurements were performed within 1 h, and the experiment results were analyzed.

### Western blotting of PI3K/AKT /mTOR signaling pathway-related proteins

After the cells in each group were harvested, lysis buffer was added to extract protein and bicinchoninic acid (BCA) method was used to measure the quantification of total protein. Then 25 μg protein was added in each well, followed by electrophoresis, transmembrane and block overnight. The membrane was washed with phosphate buffer solution tween-20 (PBST) for 5 times and added with primary antibodies (Cell Signaling Technology Inc., Beverly, MA, USA; PI3K antibody: 1: 1000; AKT antibody: 1: 1000; p-AKT antibody 1: 2000; mTOR antibody 1: 1 000; p-mTOR antibody: 1: 1 000) at 37°C for 1.5 h. After the membrane was washed for 5 times by PBS, horseradish peroxidase labeled secondary antibody was added for incubation at 37°C for 1 h. Washed in PBS for 5 times, the membrane was stained, exposed to light, developed with BIORAD GELDOC XR Gel Imaging System and fixation. Quantity One Basic software was applied for analysis of protein bands.

### Statistical analysis

SPSS 19.0 statistical software (SPSS Inc., Chicago, IL, USA) was used for statistical analysis. Count data were displayed as rates or percentages, and comparisons between two groups were conducted using chi-squared tests. Measurement data were displayed as x¯±s, and comparisons among multiple groups were conducted using one-way analysis of variance (ANOVA) (homogeneity of variance tests were conducted prior to analysis). The pair-wise comparisons of group means were conducted using LSD *t-*tests. The relationship between expressions of miR-21 and PDCD4 was analyzed using Pearson correlation analysis. Two-tailed *P* < 0.05 was considered as statistically significant.
